# Antibiotic residues and antimicrobial resistance patterns of bacteria isolated from beef at Ruti Rufura Slaughterhouse, southwestern Uganda

**DOI:** 10.3389/fmicb.2026.1952715

**Published:** 2026-09-03

**Authors:** Barbra Tuhamize, Kica Daniel

**Affiliations:** 1 Department of Microbiology and Parasitology, Faculty of Health Sciences, Mbarara University of Science and Technology, Mbarara City, Uganda; 2 SAMRC-UNIVEN Antimicrobial Resistance and Global Health Research Unit, Institute for Pathogen and Global Health Research, University of Venda, Thohoyandou, South Africa; 3 Department of Pharmacy and Pharmaceutical Sciences, Faculty of Health Sciences, Mbarara University of Science and Technology, Mbarara City, Uganda

**Keywords:** antimicrobial residues, antimicrobial resistance, beef, food safety, multidrug-resistant bacteria, one health, slaughterhouse surveillance

## Abstract

**Background:**

Antimicrobial use in livestock contributes to the emergence of antimicrobial-resistant bacteria and the persistence of antimicrobial residues in foods of animal origin. The coexistence of antimicrobial residues and resistant bacteria in beef poses a major food safety and public health concern. However, data on these hazards in Uganda remain limited. This study assessed antibiotic residues and antimicrobial resistance among bacteria isolated from beef marketed in southwestern Uganda.

**Methods:**

A cross-sectional laboratory-based study was conducted from May to August 2025 using beef samples from 92 randomly selected cattle carcasses at Ruti Rufura Slaughterhouse, Mbarara City, Uganda. Antibiotic residues were screened by agar well diffusion and confirmed for penicillin and oxytetracycline using thin-layer chromatography. Bacterial isolates were identified using standard microbiological methods, and antimicrobial susceptibility was determined by the Kirby–Bauer disk diffusion method according to CLSI guidelines.

**Results:**

Antibiotic residues were detected in 86/92 (93.5%) samples. Thin-layer chromatography confirmed penicillin residues in 84 (97.7%) and oxytetracycline residues in 71 (82.6%) samples, with 73.1% containing both antibiotics. A total of 125 bacterial isolates representing seven genera were recovered, predominantly *Proteus* spp. (30.4%), *Citrobacter* spp. (26.4%), and *Escherichia coli* (16.8%). Among 75 viable isolates, resistance was highest to ampicillin (97.3%), chloramphenicol (86.7%), tetracycline (82.7%), ceftriaxone (57.3%), and ciprofloxacin (54.7%), while gentamicin showed the highest susceptibility (86.7%). Oxytetracycline residues were significantly associated with tetracycline resistance and penicillin residues with ampicillin resistance (both *P < 0.001*).

**Conclusion:**

Beef marketed in southwestern Uganda frequently contains antimicrobial residues and antimicrobial-resistant bacteria, posing a potential food safety risk. Strengthened antimicrobial stewardship, enforcement of withdrawal periods, and integrated surveillance of antimicrobial residues and resistance in foods of animal origin are needed to support One Health efforts against antimicrobial resistance.

## Introduction

Antimicrobial agents play an essential role in the prevention and treatment of bacterial infections in food-producing animals, contributing to animal health, productivity, and food security. However, the extensive and sometimes inappropriate use of antimicrobials in livestock production has accelerated the emergence and dissemination of antimicrobial resistance (AMR), which is now recognized as one of the major global public health challenges. The global consumption of antimicrobials in food-producing animals has increased substantially over recent decades, driven by rising demand for animal protein, intensification of livestock production, and routine antimicrobial use for therapeutic and non-therapeutic purposes (Van Boeckel et al., 2015). Without effective interventions, increasing antimicrobial use in food animals is expected to continue contributing to the global burden of AMR (World Health Organization, 2026).

In addition to promoting resistance, inappropriate antimicrobial use in food animals may result in the presence of drug residues in edible animal products, including meat, milk, and eggs. Antimicrobial residues occur when withdrawal periods are not adequately observed or when antimicrobials are administered without appropriate veterinary oversight (Adegbeye et al., 2024). Consumption of animal products containing residues above recommended limits may pose risks to human health, including allergic reactions, toxic effects, disruption of normal microbiota, and exposure of bacterial populations to sub-inhibitory antimicrobial concentrations that may favor selection of resistant organisms (Beyene, 2015). The World Health Organization (WHO) has emphasized the importance of responsible antimicrobial use in food-producing animals to minimize risks to public health while preserving the effectiveness of medically important antimicrobials (Aidara-Kane et al., 2018).

Antibiotic residues in animal-derived foods remain a significant concern, particularly in low- and middle-income countries where regulatory frameworks, surveillance systems, veterinary services, and enforcement of withdrawal periods may be limited. In Uganda, reports of substantial levels of antimicrobial residues in beef from both rural and urban settings, highlighting challenges associated with antimicrobial use practices and compliance with recommended withdrawal periods (Basulira et al., 2019). Similarly, evidence from systematic reviews and studies conducted across African countries demonstrates the widespread occurrence of antimicrobial residues in animal-derived foods (Zulu et al., 2026, Simbine-Ribisse et al., 2024). This wide spread contamination reflects inappropriate antimicrobial use, poor adherence to withdrawal periods, limited farmer awareness, and weak regulatory oversight (Zulu et al., 2026, Simbine-Ribisse et al., 2024, Oladeji et al., 2025). These findings highlight the need for continued monitoring of antimicrobial residues in animal-derived foods to protect consumers and support food safety systems (Zulu et al., 2026).

Beyond residue concerns, livestock production systems represent important reservoirs for antimicrobial-resistant bacteria and resistance determinants (Tang et al., 2017). Antimicrobial exposure creates selective pressure within animal-associated microbial communities, favoring the survival and proliferation of resistant organisms (Manyi-Loh et al., 2018). These resistant bacteria and and their resistance determinants may subsequently disseminate through food products, environmental pathways, and direct contact between animals and humans (Manyi-Loh et al., 2018, Tang et al., 2017). Resistant bacteria originating from livestock may be transmitted to humans through consumption of contaminated meat, direct occupational exposure during animal handling and slaughter, or environmental dissemination via animal waste and agriculture runoff (Lekshmi et al., 2017, Robinson et al., 2016). These interconnected transmission pathways underscore antimicrobial resistance as a quintessential One Health challenge requiring coordinated interventions across the human, animal, and environmental health sectors (Van Boeckel et al., 2019, Woolhouse, 2024)

Meat products, particularly beef, provide an important interface for the transmission of antimicrobial-resistant bacteria because contamination may occur during slaughter, processing, transportation, and retail handling. Bacteria commonly associated with meat contamination include *Escherichia coli*, *Salmonella* spp., *Enterobacter* spp., and other Enterobacterales, some of which are important foodborne pathogens and reservoirs of clinically relevant resistance determinants (Barco et al., 2015). Increasing resistance among livestock-associated bacteria to critically important antimicrobials, including third-generation cephalosporins and fluoroquinolones, is of particular concern because these agents are essential for the management of severe human infections (World Health Organization, 2022). Recent studies across Africa have demonstrated considerable levels of antimicrobial resistance among livestock-associated bacteria, underscoring the contribution of food animals to the broader AMR burden (Kafaiya et al., 2025, Tufa et al., 2026, Tuhamize et al., 2023, 2025).

In Uganda, cattle production contributes significantly to livelihoods, nutrition, and economic development, with beef consumed widely across communities (FAO, 2022). However, information regarding the occurrence of antimicrobial residues and resistance profiles of bacteria associated with beef remains limited, particularly at slaughterhouse and retail levels. Existing studies have mainly focused on either antimicrobial residues or resistance among selected bacterial populations, leaving limited understanding of the relationship between antimicrobial exposure, residue occurrence, and resistance patterns in beef products. Generating such evidence is essential for guiding antimicrobial stewardship interventions, strengthening food safety surveillance, and informing One Health strategies for addressing AMR (Woolhouse, 2024, World Health Organization, 2026).

Therefore, this study aimed to determine the occurrence of antibiotic residues and characterize antimicrobial resistance patterns among bacterial isolates recovered from beef obtained from Ruti Rufura Slaughterhouse in southwestern Uganda. The findings will provide evidence on the intersection between antimicrobial use practices, food safety, and antimicrobial resistance, contributing to efforts aimed at improving surveillance and stewardship of antimicrobial use within livestock production systems.

## Materials and methods

### Study design and study area

A cross-sectional laboratory-based study was conducted to assess antibiotic residues and antimicrobial resistance patterns of bacteria isolated from beef obtained from Ruti Rufura Slaughterhouse, located in Ruti, Nyamitanga Division, Mbarara City, southwestern Uganda. The slaughterhouse is one of the major beef processing facilities supplying butcheries within Mbarara City and surrounding areas. The study was conducted during November 2025.

### Sample size determination and sampling

The sample size was determined using the Krejcie and Morgan (1970) sample size determination approach based on the average number of cattle slaughtered per day. Approximately 30 cattle were slaughtered daily, and 92 carcasses were randomly selected for inclusion in the study. Simple random sampling was employed to select carcasses during sampling visits conducted on 6th May, 3rd June, 8th July, and 12th August 2025. Carcasses available for sampling during each visit were assigned identification numbers, and selection was performed using a simple random approach to minimize selection bias. From each selected carcass, approximately equal portions of liver, kidney, and gluteal muscle were aseptically collected under aseptic conditions for laboratory analysis.

### Sample collection and transportation

Sample collection was performed following the procedure described by Ramatla et al. (2016). Fresh liver, kidney, and gluteal muscle samples were aseptically collected from selected carcasses immediately after slaughter. Samples were placed in sterile sample containers, transported in an insulated ice box under cold-chain conditions, and delivered to the Pharmaceutical Chemistry and Analytical Research Laboratory, Mbarara University of Science and Technology, for analysis.

### Preparation of beef extracts

Sample preparation was performed according to Rood et al. (2018) with minor modifications. Briefly, 5 g of each beef sample was cut into small pieces and homogenized. Ten milliliters of phosphate-buffered saline (PBS; pH 6.5) were added, followed by 2 mL of 30% trichloroacetic acid to precipitate proteins. The homogenate was centrifuged for 20 min, after which the supernatant was collected. An equal volume of diethyl ether was added to the filtrate, mixed thoroughly, and allowed to stand at room temperature for 10 min. The extracted solution was transferred into sterile test tubes, sealed, and refrigerated until analysis.

### Detection of antibiotic residues

#### Agar well diffusion assay

Antibiotic residues were initially screened using the agar well diffusion assay as described by Balouiri et al. (2016) with slight modifications. Mueller-Hinton agar plates were inoculated separately with standardized suspensions of *Staphylococcus aureus* ATCC 25923 and *Escherichia coli* ATCC 25922. These indicator organisms were selected because they represent Gram-positive and Gram-negative bacteria, respectively, and are commonly used as reference strains in antimicrobial activity and residue screening assays. Wells measuring 8 mm in diameter were aseptically prepared using a sterile cork borer, and aliquots of prepared beef extracts were dispensed into the wells. Plates were incubated aerobically at 35–37 °C for 24 h. Following incubation, the diameters of inhibition zones surrounding each well were measured in millimeters. The presence of an inhibition zone was interpreted as evidence of antimicrobial residues in the beef sample.

#### Thin layer chromatography (TLC)

Samples that tested positive in the agar well diffusion assay were further analyzed by Thin Layer Chromatography (TLC) to identify penicillin and oxytetracycline residues. These antimicrobial classes were selected for confirmation because penicillins and tetracyclines are among the commonly used veterinary antimicrobials in food-producing animals and are frequently reported as residues in animal-derived foods. Sample extracts and antibiotic reference standards were spotted onto silica gel TLC plates approximately 1 cm above the lower edge using glass capillary tubes. Plates were developed in a mobile phase consisting of methanol, acetone, and aqueous ammonia (5:5:2.5, v/v/v) inside sealed chromatography tanks. Following development, chromatograms were visualized under ultraviolet (UV) light and subsequently sprayed with ninhydrin reagent to enhance visualization of antibiotic spots.

#### Isolation and identification of bacteria

For bacteriological analysis, 1 mL of each homogenized beef sample was inoculated onto Plate Count Agar (PCA) for enumeration of viable bacteria and Eosin Methylene Blue (EMB) agar for selective isolation of Gram-negative bacteria. Plates were incubated aerobically at 37 °C for 24 h. Distinct colonies were sub cultured onto MacConkey agar to obtain pure isolates. Bacterial identification was performed using standard biochemical methods, including Simmons Citrate Agar (SCA), Triple Sugar Iron Agar (TSIA), and Sulfide-Indole-Motility (SIM) medium. These conventional biochemical methods are widely used for routine bacterial identification, particularly in settings where molecular identification platforms are not routinely available. Following incubation for 48 h, isolates were identified based on their biochemical characteristics according to standard microbiological identification procedures and the Clinical and Laboratory Standards Institute (CLSI) guidelines.

#### Antimicrobial susceptibility testing

**FIGURE 1:**
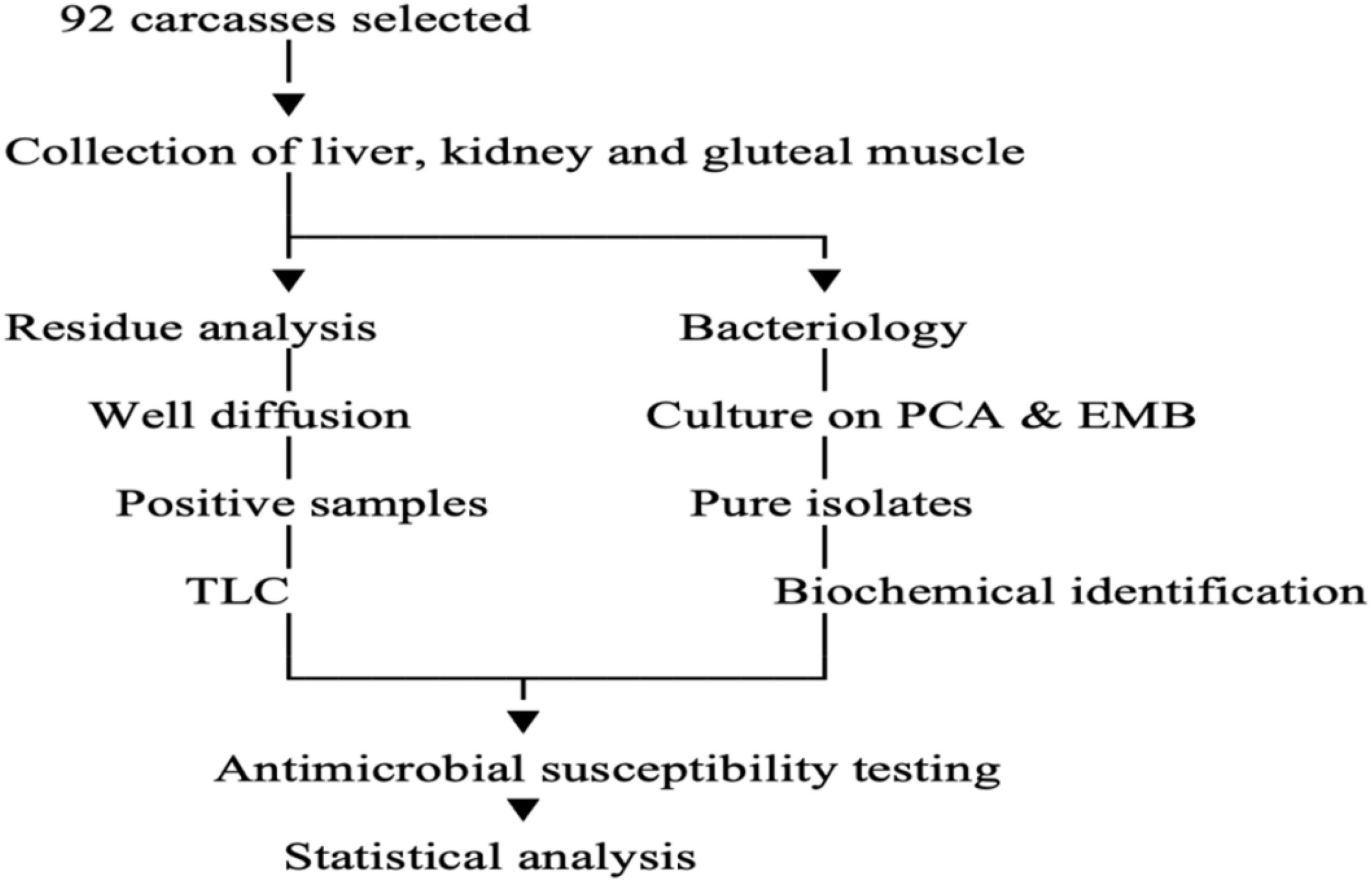
Study workflow.

Antimicrobial susceptibility testing was performed using the Kirby–Bauer disk diffusion method following the recommendations of Bauer et al. (1966) and the Clinical and Laboratory Standards Institute (CLSI) guidelines (2025). Fresh bacterial colonies were suspended in sterile saline and adjusted to the turbidity of a 0.5 McFarland standard. A 50 μL aliquot of each suspension was uniformly spread over Mueller-Hinton agar plates using sterile cotton swabs. After allowing the inoculum to dry for approximately 5 min, antibiotic discs were placed on the agar surface. The antimicrobial agents evaluated included: Ampicillin (10 μg), Ciprofloxacin (5 μg), Ceftriaxone (30 μg), Gentamicin (10 μg), Chloramphenicol (30 μg), Tetracycline (30 μg). The plates were incubated aerobically at 37 °C for 24 h, after which inhibition zone diameters were measured in millimeters. Isolates were classified as susceptible, intermediate, or resistant according to CLSI interpretive criteria. For data analysis, isolates categorized as intermediate were grouped with resistant isolates because reduced susceptibility may limit the clinical utility of these antimicrobial agents. This approach was considered appropriate for surveillance purposes, although it may result in higher estimates of resistance prevalence. The overall study flow is presented in (Figure 1).

### Data analysis

Data were entered into Microsoft Excel and analyzed using IBM SPSS Statistics (version 26, IBM Corp., Armonk, NY, USA). Descriptive statistics were used to summarize the prevalence of antibiotic residues, bacterial isolates, and antimicrobial resistance patterns. Categorical variables were expressed as frequencies and percentages. Differences in the occurrence of antibiotic residues between indicator organisms and among beef tissues were assessed using the Chi-square test. The relationship between antibiotic residues and corresponding antimicrobial resistance patterns was also evaluated using the Chi-square test. Descriptive statistics and Chi-square tests were selected to summarize occurrence patterns and assess associations among categorical variables, considering the exploratory nature of the study and available sample size. Statistical significance was considered at *P* < 0.05.

## Results

### Detection of antibiotic residues in beef

**FIGURE 2:**
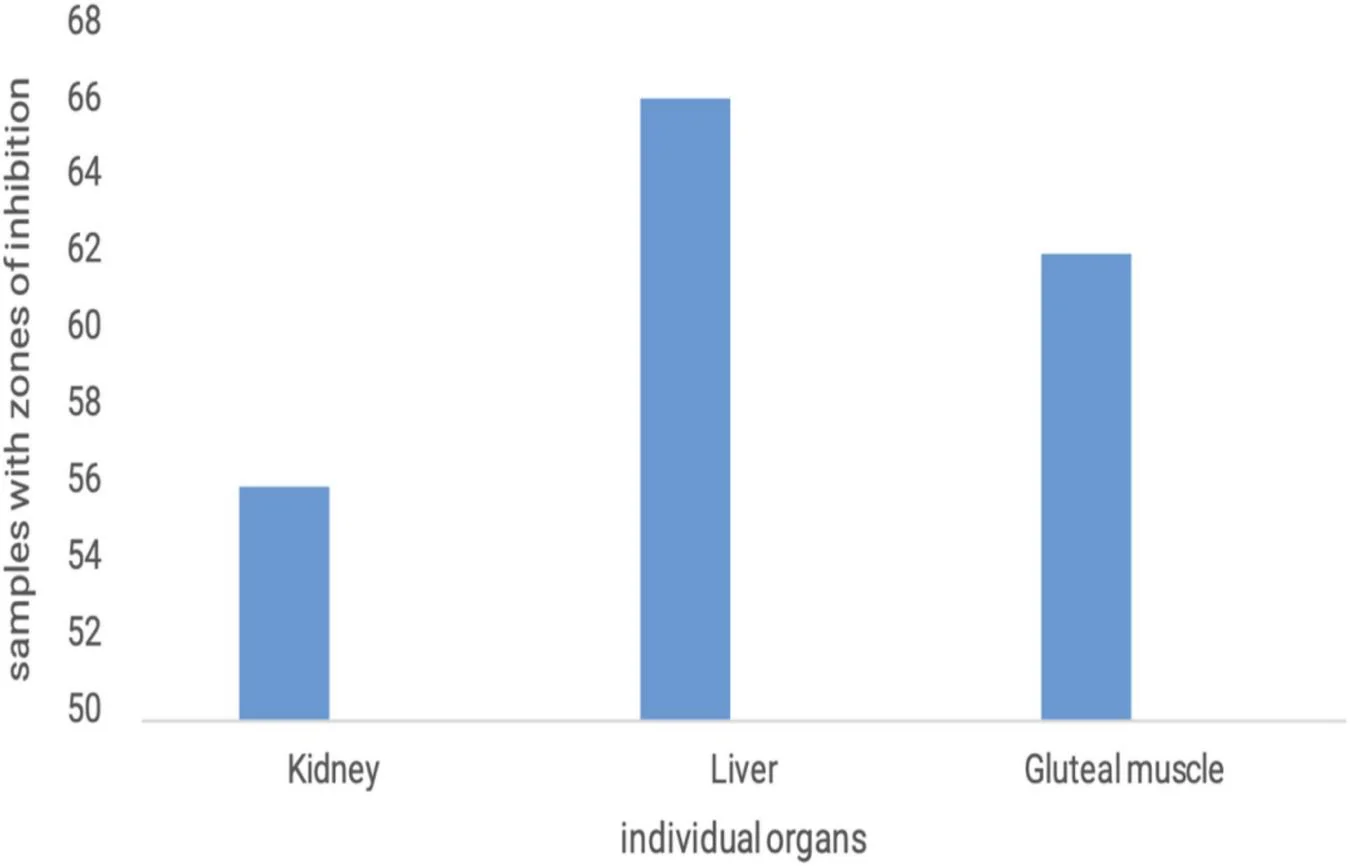
Distribution of inhibition zones indicating antibiotic residues in liver, kidney, and gluteal muscle.

A total of 92 beef samples collected from butcheries supplied by Ruti Rufura Slaughterhouse were screened for antibiotic residues using agar well diffusion assay. Overall, 86/92 (93.5%) samples tested positive for antibiotic residues. Detection using *Staphylococcus aureus* and *Escherichia coli* indicator organisms yielded comparable results, with no statistically significant difference *(P = 0.646)*. Organ-specific analysis showed that liver samples had the highest frequency of residues, followed by gluteal muscle and kidney, although these differences were not statistically significant (*P > 0.05*) (Figure 2).

### Identification of antibiotic residues by thin layer chromatography

Samples positive by microbiological screening were subjected to TLC for identification of specific antibiotic classes. Penicillin residues were detected in 84 (97.8%) samples, while oxytetracycline residues were identified in 71 (82.6%). Approximately 73% of samples contained both antibiotics simultaneously. Penicillin residues predominated across all organs, whereas liver samples exhibited the highest frequency of oxytetracycline residues (Table 1).

**TABLE 1:** Detection of penicillin and oxytetracycline residues in beef samples.

Residue	Overall (%)	Kidney	Liver	Muscle
Penicillin	97.8	95.3	76.7	81.4
Oxytetracycline	82.6	52.3	59.3	38.4
Both residues	73.1	47.7	54.7	29.0

### Bacterial isolates recovered from beef

Culture and biochemical identification yielded seven bacterial genera from beef samples. *Proteus spp*. (30.4%) and *Citrobacter spp*. (26.4%) were the predominant isolates, accounting for approximately one-third of all isolates each, followed by *Escherichia coli* (16.8%), *Enterobacter spp*. (12.8%), and *Salmonella spp.* (8.8%). The least frequently isolated bacteria were *Providencia alcalifaciens* and *Pseudomonas aeruginosa*, each accounting for 2.4% of the total isolates (Table 2).

**TABLE 2:** Number and percentage of bacterial isolates recovered from liver, kidney, and gluteal muscle samples.

Microorganism	Kidney, *n* (%) (*n* = 48)	Liver, *n* (%) (*n* = 39)	Gluteal muscle, *n* (%) (*n* = 38)	Total, *n* (%) (*N* = 125)
*Citrobacter* spp.	8 (16.7)	9 (23.1)	16 (42.1)	33 (26.4)
*Enterobacter* spp.	5 (10.4)	6 (15.4)	5 (13.2)	16 (12.8)
*Salmonella* spp.	2 (4.2)	6 (15.4)	3 (7.9)	11 (8.8)
*Proteus* spp.	16 (33.3)	13 (33.3)	9 (23.7)	38 (30.4)
*Escherichia coli*	14 (29.2)	2 (5.1)	5 (13.2)	21 (16.8)
*Pseudomonas aeruginosa*	1 (2.1)	2 (5.1)	0 (0.0)	3 (2.4)
*Providencia alcalifaciens*	2 (4.2)	1 (2.6)	0 (0.0)	3 (2.4)
Total	48 (100.0)	39 (100.0)	38 (100.0)	125 (100.0)

### Antimicrobial susceptibility profiles

Of the 125 bacterial isolates recovered from beef samples, 75 isolates that remained viable following preservation and were successfully recovered for testing were subjected to antimicrobial susceptibility testing. Intermediate susceptibility results were categorized as resistant for analysis. High levels of resistance were observed among the tested isolates, with the highest resistance recorded against ampicillin (97.3%), followed by chloramphenicol (86.7%) and tetracycline (82.7%). Resistance to ceftriaxone and ciprofloxacin was 57.3% and 54.7%, respectively. Gentamicin demonstrated the highest activity against the isolates, with 86.7% of isolates classified as susceptible and 13.3% resistant. Overall, the findings demonstrate a high burden of antimicrobial resistance among bacterial isolates recovered from beef samples (Table 3).

**TABLE 3:** Antimicrobial susceptibility profiles of isolates from beef obtained from the slaughterhouse.

Antibiotic	Susceptible (%)	Resistant (%)
Ampicillin	2.7% (2/75)	97.3% (73/75)
Ciprofloxacin	45.3% (34/75)	54.7% (41/75)
Ceftriaxone	42.7% (32/75)	57.3% (43/75)
Gentamicin	86.7% (65/75)	13.3% (10/75)
Chloramphenicol	13.3% (10/75)	86.7% (65/75)
Tetracycline	17.3% (13/75)	82.7% (62/75)

### Association between antibiotic residues and antimicrobial resistance

A significant association was observed between the occurrence of antibiotic residues and phenotypic antimicrobial resistance. The prevalence of oxytetracycline residues corresponded closely with resistance to tetracycline, while penicillin residues closely mirrored ampicillin resistance. Both relationships were statistically significant (*P < 0.001*), indicating an association between the presence of antimicrobial residues and corresponding resistance phenotypes among bacterial isolates (Table 4).

**TABLE 4:** Relationship between antimicrobial residues and antimicrobial resistance among bacterial isolates from beef samples.

Antimicrobial class	Antimicrobial residue detected	Prevalence of antimicrobial residue	Corresponding antimicrobial resistance	Prevalence of resistance	Association (*p*-value)
Tetracyclines	Oxytetracycline (OTC) residue	82.6%	Tetracycline resistance	82.6%	*p* < 0.001
Penicillins	Procaine penicillin residue	97.8%	Ampicillin resistance	97.3%	*p* < 0.001

## Discussion

This study provides evidence of widespread occurrence of antibiotic residues and antimicrobial-resistant bacteria in beef obtained from Ruti Rufura Slaughterhouse, southwestern Uganda. The detection of antibiotic residues in 93.5% of the analyzed beef samples suggests possible non-adherence to recommended antimicrobial withdrawal periods and indicates possible exposure of consumers to antimicrobial residues through consumption of animal products. However, because this study employed qualitative screening methods, it cannot determine whether residue concentrations exceeded established Maximum Residue Limits (MRLs). These findings are consistent with previous reports from Uganda and other African countries demonstrating the occurrence of antibiotic residues in beef and other animal-derived foods, largely attributed to inappropriate antimicrobial use and non-compliance with recommended withdrawal periods (Basulira et al., 2019, Olatoye and Ehinmowo, 2010, Beyene, 2015). The presence of antimicrobial residues in food animals is a recognized global food safety concern because inappropriate antimicrobial use and failure to observe withdrawal periods may result in residues entering the food chain, increasing the risk of adverse health effects and contributing to the development and dissemination of antimicrobial resistance (Zulu et al., 2026). However, quantitative analytical methods are required to determine compliance with regulatory MRLs and accurately assess potential consumer exposure.

The detection of penicillin residues in 97.8% of samples and oxytetracycline residues in 82.6% of samples demonstrates the frequent use of these antimicrobial classes in cattle production within the study area. Penicillins and tetracyclines are among the most widely used veterinary antimicrobial classes globally because of their broad-spectrum activity, affordability, and widespread availability, particularly in food-producing animals (Van Boeckel et al., 2015, 2019; Aidara-Kane et al., 2018). Similar studies across sub-Saharan Africa have reported substantial antimicrobial residues in meat products, indicating that antimicrobial residues remain a widespread food safety concern in the region. These findings have been attributed to indiscriminate antimicrobial use, inadequate veterinary oversight, limited farmer awareness of withdrawal periods, and weak enforcement of regulations governing veterinary drug use (Beyene, 2015). Although our findings demonstrate frequent detection of these residues, the concentrations were not quantified; therefore, conclusions regarding compliance with established Maximum Residue Limits cannot be drawn.

The simultaneous detection of both penicillin and oxytetracycline residues in 73.1% of samples further indicates repeated exposure of cattle to multiple antimicrobial agents before slaughter. Similar findings have been reported in food-producing animals, where concurrent use of different antimicrobial classes reflects therapeutic practices aimed at treating diverse bacterial infections or empirical administration without adequate veterinary oversight (Manyi-Loh et al., 2018, Beyene, 2015). Although oxytetracycline residues were detected more frequently in liver samples than in kidney and muscle tissues, these differences were not statistically significant. Nevertheless, the observed distribution is biologically plausible because the liver plays a central role in drug metabolism and biotransformation, while the kidneys are primarily responsible for the excretion of many drugs and their metabolites, leading to differential residue accumulation among edible tissues (Olatoye and Ehinmowo, 2010, FAO, 2022). From a food safety perspective, the widespread detection of antimicrobial residues across all edible tissues warrants further investigation. Because residue concentrations were not quantified, this study cannot determine compliance with established Maximum Residue Limits or directly assess consumer risk. Nevertheless, these findings underscore the need for improved antimicrobial stewardship, better adherence to withdrawal periods, and confirmatory quantitative residue monitoring (Aidara-Kane et al., 2018).

Bacteriological analysis revealed recovery of diverse bacterial genera, including *Proteus spp.*, *Citrobacter spp.*, *Escherichia coli*, *Enterobacter spp*., and *Salmonella spp*. The predominance of *Proteus spp*. (30.4%) and *Citrobacter spp*. (26.4%) suggest contamination of beef products from intestinal contents, slaughterhouse environments, contaminated equipment, or handling practices. However, the occurrence of these organisms may not only reflect contamination events but also their ecological adaptability and ability to persist in diverse environments, including soil, water, animal-associated environments, and processing facilities. Their recovery from beef products may therefore reflect the complex microbial ecology of slaughterhouses, where enteric organisms can be introduced, maintained, and redistributed through animal handling, processing surfaces, and environmental exposure. These enteric organisms are frequently reported in meat products and reflect the influence of slaughter hygiene, processing conditions, and post-slaughter handling on microbial quality and food safety (Kafaiya et al., 2025, Ababu et al., 2026; Lekshmi et al., 2017, Tufa et al., 2026). The recovery of potential foodborne pathogens such as *E. coli* and *Salmonella* is particularly important because these organisms are major causes of foodborne infections and may act as reservoirs for antimicrobial resistance determinants that can be transmitted through the food chain (Authority, 2025).

The antimicrobial susceptibility findings demonstrate a high burden of resistance among bacterial isolates recovered from beef samples. Resistance was highest against ampicillin (97.3%), chloramphenicol (86.7%), and tetracycline (82.7%), whereas lower resistance was observed against gentamicin (13.3%). The predominance of resistance to beta-lactams and tetracyclines may be associated with the widespread use of these antimicrobial classes in food animal production, which creates selection pressure favoring resistant bacterial populations. Similar resistance patterns among livestock-associated bacteria have been reported across several African countries, highlighting food animals as important reservoirs and potential vehicles for dissemination of antimicrobial-resistant organisms through the food chain (Manyi-Loh et al., 2018, Tufa et al., 2026).

The observed resistance to ceftriaxone (57.3%) is of particular concern because third-generation cephalosporins are classified as critically important antimicrobials for human medicine and are widely used for the treatment of serious infections. Resistance to these agents threatens the effectiveness of important therapeutic options and represents a major One Health concern (GBD 2021 Antimicrobial Resistance Collaborators, 2024, Tang et al., 2017, FAO, 2022). Resistance to third-generation cephalosporins among food-associated Enterobacterales is also epidemiologically important because it may indicate the circulation of organisms carrying clinically relevant resistance mechanisms, including extended-spectrum beta-lactamase (ESBL) production, although ESBL determinants were not investigated in this study. Therefore, surveillance of third-generation cephalosporin resistance in food-producing animals remains an important component of integrated One Health AMR monitoring. Although ceftriaxone use in veterinary systems may vary, exposure of livestock-associated bacteria to antimicrobial agents can select for resistant populations, which may contribute to the dissemination of resistance determinants through the food chain, environmental contamination, and direct animal-human contact (Robinson et al., 2016). Similarly, resistance to ciprofloxacin (54.7%) is concerning because fluoroquinolones are classified among the highest priority critically important antimicrobials by WHO due to their importance in treating severe human infections. The occurrence of ciprofloxacin-resistant bacteria in beef products highlights the potential role of the food chain in the dissemination of clinically important resistance determinants, particularly where resistant organisms originating from livestock may circulate between animals, humans, and the environment (World Health Organization, 2022). These findings support the inclusion of food-animal production systems within national and global AMR surveillance frameworks, where monitoring of resistance trends across animal, human, and environmental sectors is essential for understanding transmission pathways and informing antimicrobial stewardship interventions.

The association observed between oxytetracycline residues and tetracycline resistance, as well as between penicillin residues and ampicillin resistance, indicates a relationship between antimicrobial residue detection and corresponding resistance phenotypes. Although antimicrobial exposure is recognized as an important driver of antimicrobial resistance, the cross-sectional design of this study does not allow causal inference regarding whether residue exposure directly contributed to the observed resistance patterns. This selective pressure can enrich resistant bacteria and promote the maintenance and horizontal transfer of antimicrobial resistance genes within livestock-associated microbial communities (Tang et al., 2017, Van Boeckel et al., 2015, FAO, 2022, McEwen and Fedorka-Cray, 2002, Marshall and Levy, 2011). These findings may reflect shared antimicrobial use practices and selection pressures within livestock production systems; however, longitudinal studies are required to establish temporal relationships and better evaluate causal pathways. Furthermore, the detection of antimicrobial residues and resistant bacteria in the same samples does not necessarily imply that the detected residues directly selected for the observed resistance phenotypes. Other factors, including historical antimicrobial exposure, antimicrobial use practices on farms, environmental contamination, and transmission of resistant organisms within livestock production systems, may also influence the occurrence of antimicrobial resistance.

The coexistence of antimicrobial residues and antimicrobial-resistant bacteria in beef represents an important One Health concern at the interface of animal health, food safety, and public health. Beef contaminated with resistant bacteria may facilitate the dissemination of antimicrobial resistance through food consumption, occupational exposure during slaughter and meat processing, and environmental contamination associated with animal production systems (Robinson et al., 2016, FAO, 2022). Although this study did not quantify antimicrobial residue concentrations or evaluate compliance with regulatory Maximum Residue Limits, the frequent detection of antimicrobial residues together with the high prevalence of antimicrobial-resistant bacteria highlights the need for strengthened antimicrobial stewardship, improved residue monitoring, and enhanced food safety practices across the livestock production chain.

## Limitations

This study has several limitations that should be considered when interpreting the findings. First, antibiotic residues were detected using agar diffusion screening and thin-layer chromatography, which identify the presence of selected residues but do not provide quantitative concentrations. Therefore, the study could not determine compliance with Maximum Residue Limits (MRLs) or directly estimate consumer exposure risks. Future studies using confirmatory quantitative approaches such as LC-MS/MS are warranted.

Second, the cross-sectional design and single-slaughterhouse sampling approach limit the ability to establish temporal relationships or generalize findings beyond the study facility. The results should therefore be interpreted as a snapshot of antibiotic residues and antimicrobial resistance patterns during the study period.

Finally, antimicrobial susceptibility testing was performed on 75 of the 125 recovered isolates because some isolates could not be successfully revived after preservation. This may have introduced potential selection bias if non-recovered isolates differed in their resistance profiles; however, this could not be assessed retrospectively.

Despite these limitations, this study provides important baseline evidence on the occurrence of antimicrobial residues and antimicrobial-resistant bacteria in beef from southwestern Uganda.

## Conclusion

This study demonstrates widespread occurrence of antimicrobial residues and antimicrobial-resistant bacteria in beef marketed in southwestern Uganda. The frequent detection of penicillin and oxytetracycline residues, together with the recovery of enteric bacteria and foodborne pathogens, highlights potential challenges in antimicrobial use practices, adherence to recommended withdrawal periods, and slaughter hygiene. However, Because residue concentrations were not quantified, these findings should not be interpreted as evidence that residue levels exceeded established Maximum Residue Limits (MRLs) or that the beef posed a direct consumer health risk.

The high levels of antimicrobial resistance observed among beef-associated bacterial isolates, including resistance to critically important antimicrobials such as ceftriaxone and ciprofloxacin, further emphasize the potential role of beef products as vehicles for dissemination of antimicrobial resistance within a One Health context. The observed association between antimicrobial residues and resistance phenotypes indicates coexistence of antimicrobial exposure indicators and resistant bacterial populations; however, this cross-sectional study cannot establish causal relationships. Strengthening antimicrobial stewardship, improving adherence to withdrawal periods, enhancing slaughter hygiene, and establishing routine surveillance of antimicrobial residues and resistance in animal-derived foods are recommended to support food safety and preserve the effectiveness of essential antimicrobials.

Strengthening antimicrobial stewardship in livestock production, improving farmer and veterinary awareness on responsible antimicrobial use and withdrawal periods, enhancing slaughter hygiene practices, and establishing routine surveillance systems for antimicrobial residues and antimicrobial resistance in animal-derived foods are recommended to support evidence-based food safety monitoring and preserve the effectiveness of essential antimicrobials.

## Data Availability

The original contributions presented in the study are included in the article/supplementary material, further inquiries can be directed to the corresponding author.
